# Supplementary material for: Temperature-triggered in situ forming lipid mesophase gel for local treatment of ulcerative colitis
Source: Nat Commun. 2023 Jun 13;14:3489. doi: 10.1038/s41467-023-39013-3 (PMC10264425; doi:10.1038/s41467-023-39013-3)
Supplement: Supplementary file 1 — Supplementary Information [file 41467_2023_39013_MOESM1_ESM.pdf]

Supplementary information

Temperature-triggered *in situ* forming gel for local treatment of ulcerative colitis

Marianna Carone[1§], Marianne R. Spalinger[2§], Robert A. Gaultney[3§], Raffaele Mezzenga[4], Kristýna Hlavačková[3], Aart Mookhoek[3], Philippe Krebs[3], Gerhard Rogler[2*], Paola Luciani[1*], Simone Aleandri[1*]*

[1] Department of Chemistry, Biochemistry and Pharmaceutical Sciences, University of Bern, Bern, Switzerland

[2] University Hospital Zurich, Clinic of Gastroenterology and Hepatology, Zurich, Switzerland

[3] Institute of Tissue Medicine and Pathology, University of Bern, Bern, Switzerland

[4] Laboratory of Food & Soft Materials, Institute of Food, Nutrition and Health, IFNH; Department for Health Sciences and Technology, D-HEST, ETH Zurich, Switzerland

**Correspondence to be sent to: E-mail: philippe.krebs@unibe.ch; gerhard.rogler@usz.ch; paola.luciani@unibe.ch; simone.aleandri@unibe.ch*

[§]These authors contributed equally

Reversibility of the transition.

Small-angle X-ray scattering (SAXS) was used to determine the lipid phase, and thus the reversibility of the transition.

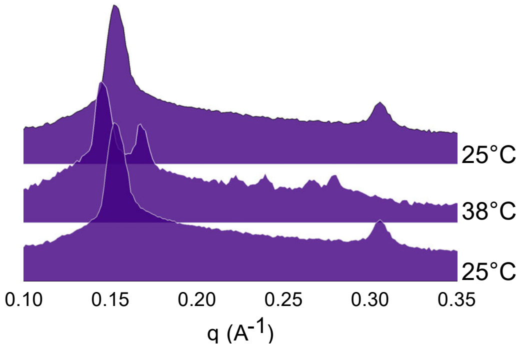

Supplementary figure 1. In vitro characterizations of the TIF-Gel: SAXS spectra acquired at different temperatures: at 25 °C (bottom), after 30 minutes equilibration at 38 °C (middle) and after 30 minutes equilibration at 25 °C (top). Source data are provided as a Source Data file.

Amplitude sweep experiments.

A stress-controlled rheometer (Modular Compact Rheometer MCR 72 from Anton Paar, Graz, Austria) was used in cone-plate geometry, 0.993° angle and 49.942 mm diameter. The temperature control was set either at 25 or 38 °C. An amplitude sweep was performed at 1 Hz between 0.002 and 100% strain to determine the linear viscoelastic regime (LVR), the yield and flow points.

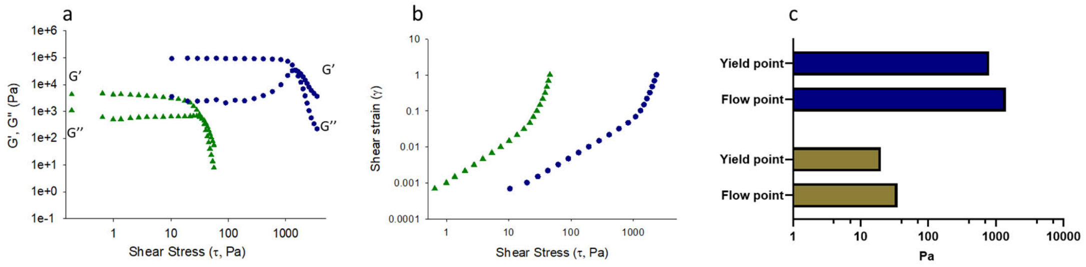

Supplementary figure 2. Amplitude sweep experiments acquired at 25°C (green symbols and bars) and at 38°C (blue symbols and bars) on empty gel. a) Storage moduli (G') and loss of moduli (G'') are plotted versus the shear stress. The yield point is the value of the shear stress at the limit of the LVE region while the flow point is the value of the shear stress at the crossover point G' = G". b) Shear strain is plotted versus the stress, and the yield point is exceeded at the point where the deformations start to deviate from linearity. c) The bar plots (also present in the main text as panel e in Figure 1) summarize the yield and flow point obtained from the amplitude sweep experiments. Source data are provided as a Source Data file.

Phase transition identification after in vivo applications.

Healthy animals were administered with 100 mL of TIF-Gel and either the excreted gel (with stool after 30 min) or the residual gel present in the colon after 6 h was collected and analyzed by SAXS (the animal was sacrificed, colon harvested and the residual gel washed 3x with PBS before analysis). As shown in Fig. S3, the Bragg reflections characteristic of L phase were present before administration at 25 °C, whereas the gel excreted with the stool showed the L→ Ia3d transition. Moreover, the lamellar phase absorbed heat and water during the experiment reaching a cubic (pn3m) phase as was already observed in the in vitro investigations.

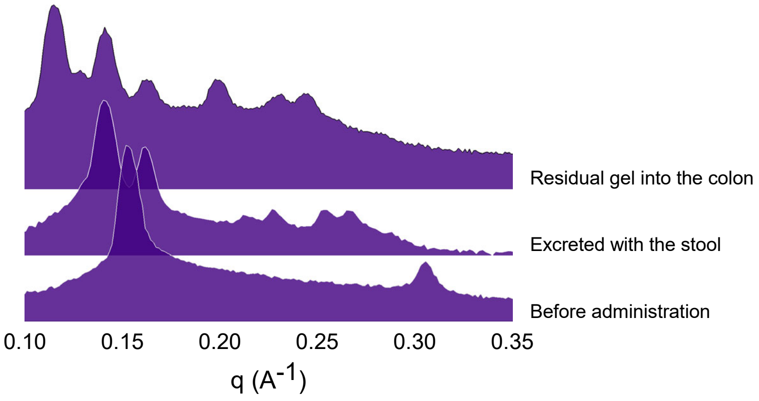

Supplementary figure 3. In vivo characterization of the TIF-Gel: SAXS spectra acquired at different time points (before administration, excreted with the stool, and the residual gel present in the colon) at 38 °C. Source data are provided as a Source Data file.

Drug homogeneity into the gel structures.

Both drugs are dissolved into the gel matrix and they do not form crystals once incorporated into the lipidic gel (at least at the drug concentrations used in this study), as proven by the absence of reflections associated with a drug crystallization in the WAXS spectra at high q (see Figure S4, panel a). We carried out additional experiments to assess whether both drugs were homogeneously distributed into the gel matrix. To determine this, the gel (loaded with TAC or TOFA) was prepared as described in the manuscript and transferred into a 2 mL Eppendorf tube. The tube was centrifuged and kept at rest for 24 hours. Subsequently, the gel was divided into 3 different layers (Top, Middle, and Bottom), and the drug content evaluated in each. As shown in Figure S4 (panel b), each layer contains the same drug amount, confirming that TOFA and TAC were homogeneously distributed.

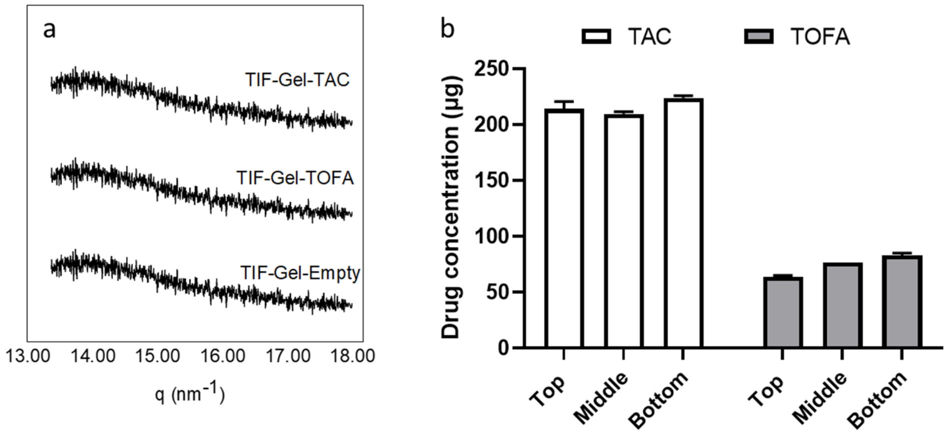

Supplementary figure 4. Drug distribution in TIF-Gel. a) WAXS spectra obtained for empty gel (bottom), TOFA loaded gel (middle) and TAC loaded-gel (top). All the WAXS spectra (acquired for 30 minutes at 25 °C) show only a broad shoulder (and no evident peak) which indicates the amorphous state of the lipidic chain and the absence of crystalline structures. b) Homogeneity of drugs in the gel. The amount of drug present in 3 different gel layers (Top, Middle and Bottom) was evaluated by HPLC. Panel b indicates mean ± SD (n= 3). Source data are provided as a Source Data file.

Gels' phase identity with 10 % w/w amounts of drugs.

Measurements were performed on a Bruker AXS Micro, as described in the main text. MLO was used as the lipid component of the mesophases and mixed with weighed amounts of drugs (10% w/w) in sealed Pyrex tubes and alternatively centrifuging (10 minutes, 5000 g) several times at room temperature until a homogenous mixture was obtained. The mesophase was then equilibrated for 48 h at room temperature.

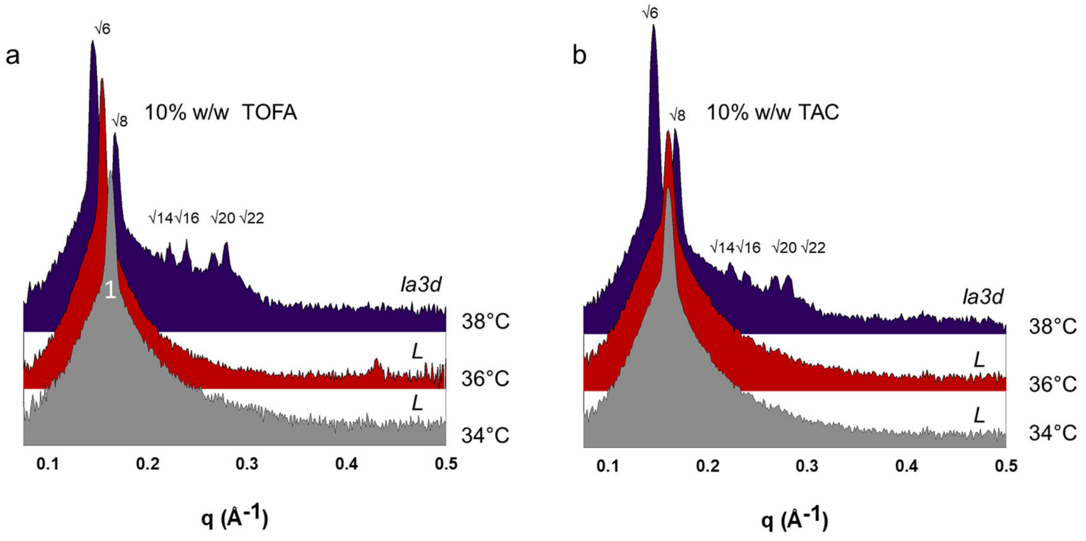

Supplementary figure 5. SAXS spectra acquired at different temperatures on gels containing 10% w/w of TOFA (a) and 10% w/w of TAC (b). Source data are provided as a Source Data file.

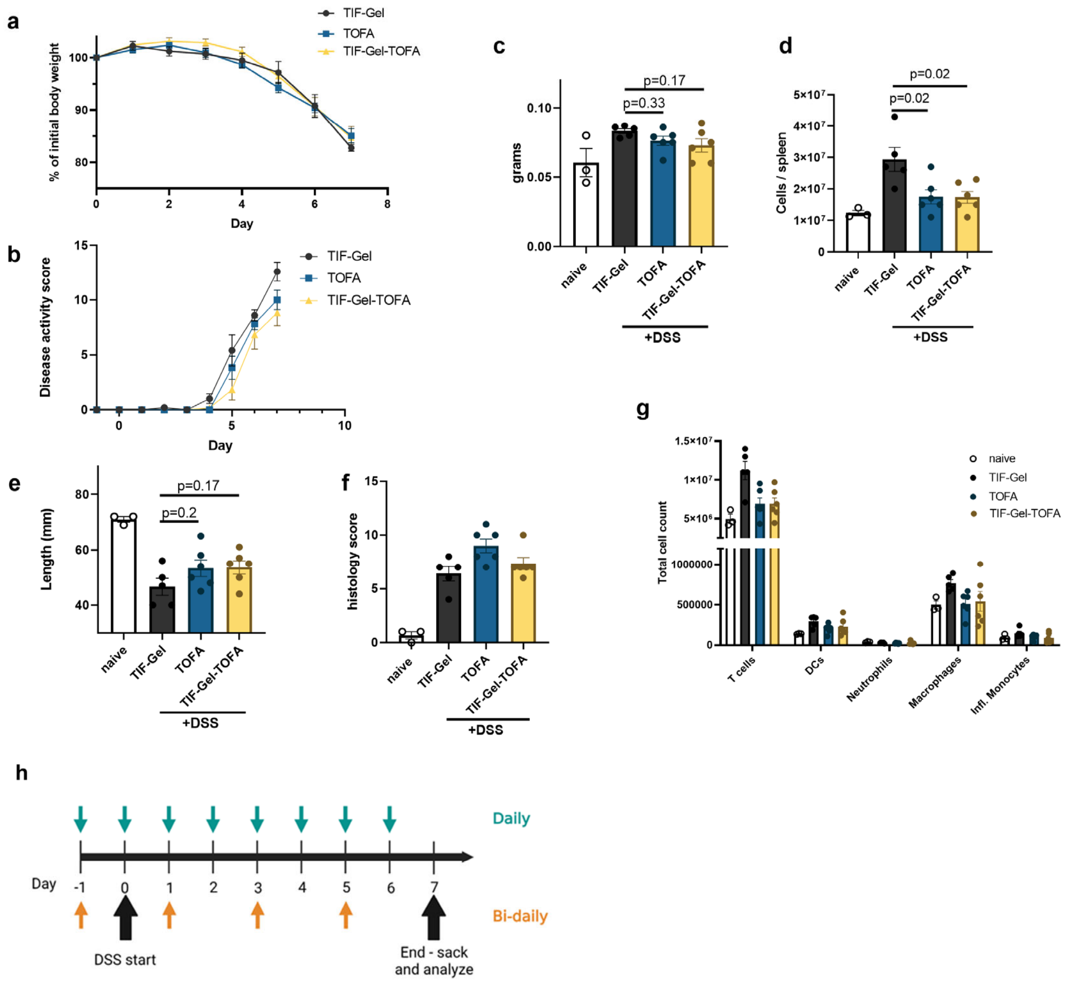

Supplementary figure 6. Daily application of TIF-Gel-TOFA did not improve animal health compared to TOFA in vehicle. Mice were treated rectally with empty gel (TIF-gel; n = 5), TOFA in vehicle (TOFA; n = 6) or drug-loaded gel (TIF-Gel-TOFA, n = 6) daily from 1 day before the start of DSS treatment until day 6). During the treatment, mice were weighed (a), and the severity of their illness was assessed (b). At day 7, mice were euthanized, and various disease parameters were recorded included spleen weight and cellularity (c and d), colon length and pathology (e and f), and the total populations of various immune cells from spleens were calculated (g). h) An application scheme detailing the timeline for the daily and bi-daily rectal applications of the various compounds. Statistical values were calculated by one-way ANOVA (c, d, e, f), multiple T tests per group with Holm-Sidak correction (g) or two-way ANOVA (a and b). *: $p<0.05$, where no value is indicated, $p>0.05$). Naïve values were excluded from analyses. Error bars are ± SEM. Source data are provided as a Source Data file.

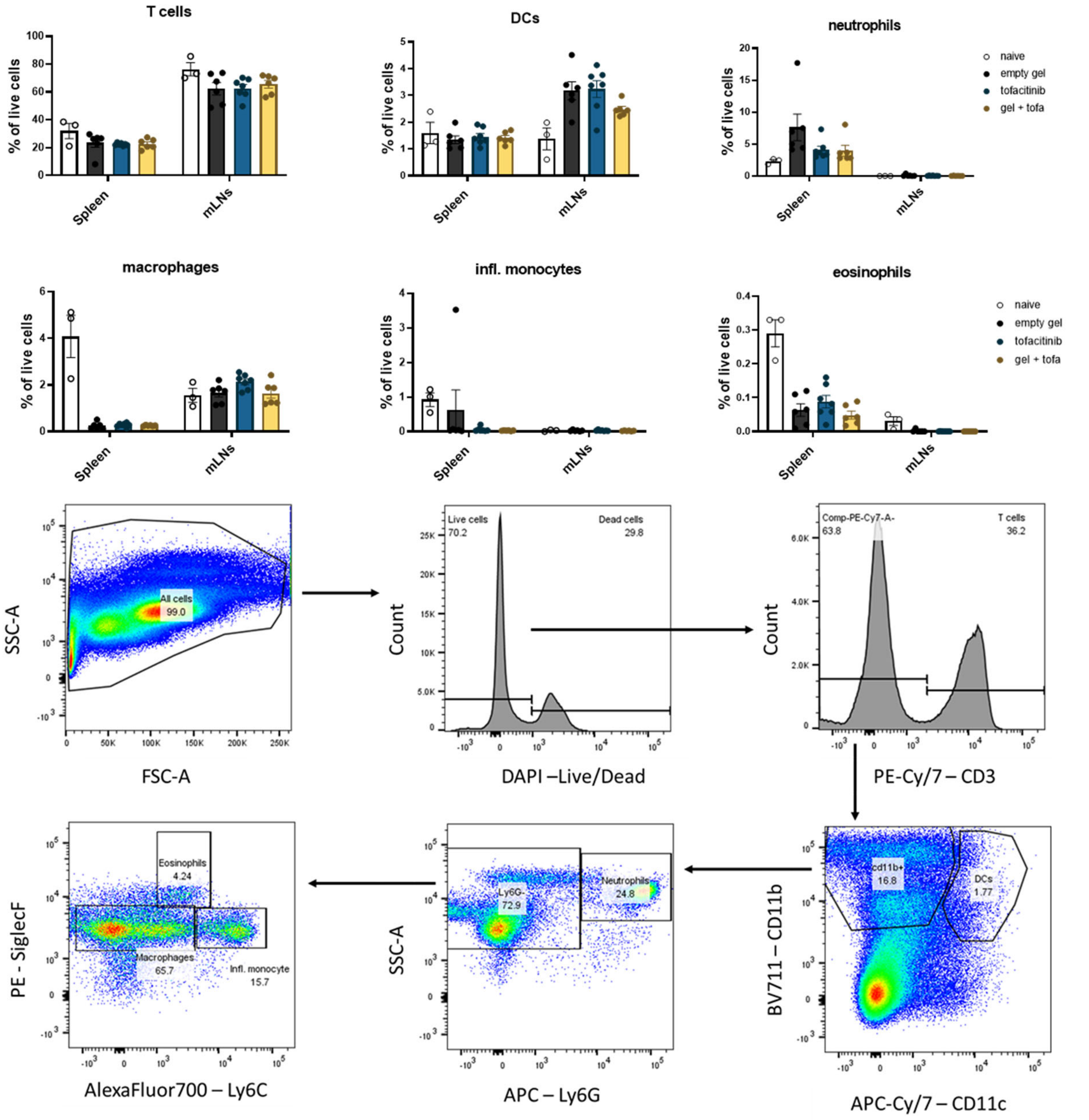

Supplementary figure 7. TIF-gel-TOFA does not rescue the changes to cell frequencies of spleen and mLN in DSS-treated mice. The relative numbers of various cell types in mouse spleens and mesenteric lymph nodes (mLNs) were quantified. Mouse numbers are the same as in main text figure 3. No statistically significant differences in percentages were observed between the different treatment groups. Data were analyzed using one-way ANOVAs. DCs, dendritic cells. Error bars are ± SEM. The gating strategy used is also depicted. Source data are provided as a Source Data file.

In vivo/ex vivo experiments to evaluate the adhesion of the TIF-Gel to the colon wall.

For *in vivo* adhesion testing, healthy animals (n= 11) received an enema of 100 µl DiR (1,1'-dioctadecyl-3,3,3',3'-tetramethylindotricarbocyanine iodide) loaded gel (DiR-TIF-Gel) under anesthesia as described in the experimental section. Animals were sacrificed after 30 minutes (n= 3), 2 and 6 hours (n=4). The distal 3 cm of the colon (including the rectum) was harvested and freshly imaged after a gentle washing with PBS. Intensity of the fluorescent signal was measured using the IVIS SpectrumCT *In Vivo* Imaging System (PerkinElmer, MA, US). DiR fluorescent signal (excitation 754 nm, emission 778 nm) was detected in the distal part of dissected colons at 3 time points post gel injection. An untreated control mouse was included in each measurement (n=3). Acquired images were analysed using the Living Image® software (PerkinElmer, MA, US). Backgrounds (untreated tissue samples) were measured for each time point. Obtained signal was analysed as radiant efficiency (RE), which is a calibrated unit that compensates for device settings and non-uniform light excitation pattern.

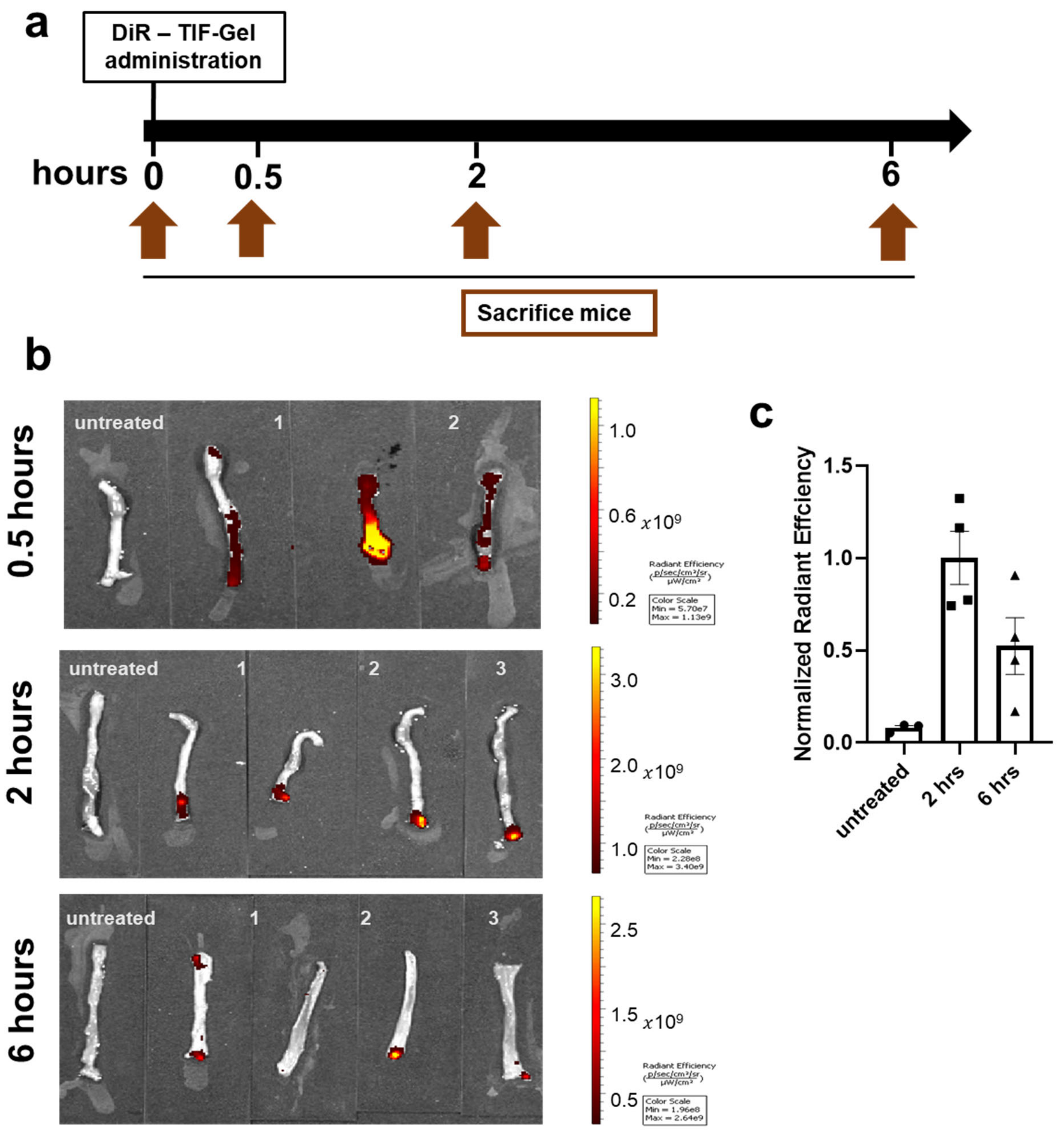

Supplementary figure 8. The TIF-Gel adheres to healthy colonic tissue for at least 6 hours. a) Experimental scheme: Healthy animals received an enema of 100 µL of DiR-TIF-Gel. Animals were sacrificed after 30 minutes (n= 3), 2 and 6 h (n= 4), and the colon was harvested and imaged (b). c) The obtained signal was analysed as radiant efficiency (RE), which was normalized to radiant efficacy recorded at 30 minutes. Graphs indicate mean normalized RE ± SEM. Source data are provided as a Source Data file.

HPLC method: Tofacitinib Citrate

Tofacitinib citrate (TOFA) was detected by reverse-phase liquid chromatography using a Macherey-Nagel Nucleosil 100-5 C18 (4.0 x 250 mm; 5.0 µm particle size) column. The mobile phase consisted of acetonitrile/methanol/water (13:13:74 v/v) + 0.1% trifluoroacetic acid at a flow rate of 1 mL/minutes, temperature 25 °C and UV detection at λ = 278 nm. An internal standard (caffeine, 20 µg/mL) was added to each sample to correct for inter-injection variation and UV detection at λ = 278 nm. Data were collected and analyzed using the software Chromeleon 7 (Thermo Fisher).

HPLC method: Tacrolimus

Tacrolimus (TAC) was detected by reverse-phase liquid chromatography using a Macherey-Nagel Nucleosil 100-5 C18 (4.0 x 250 mm; 5.0 µm particle size) column. The mobile phase consisted of methanol/water (80:20 v/v) + 0.1% trifluoroacetic acid at a flow rate of 1 mL/minutes, temperature 50 °C and UV detection at λ = 214 nm. An internal standard (ketoconazole, 20 µg/mL) was added to each sample to correct for inter-injection variation and UV detection at λ = 278 nm. Data were collected and analyzed using the software Chromeleon 7 (Thermo Fisher).

Stability Study of TAC and TOFA

The stability of the drugs – TOFA and TAC – was monitored over one month. At specific time points, an aliquot of the formulation was analyzed at the HPLC, and the content of the drug recorded. Data are expressed as relative percentage referred to day 0.

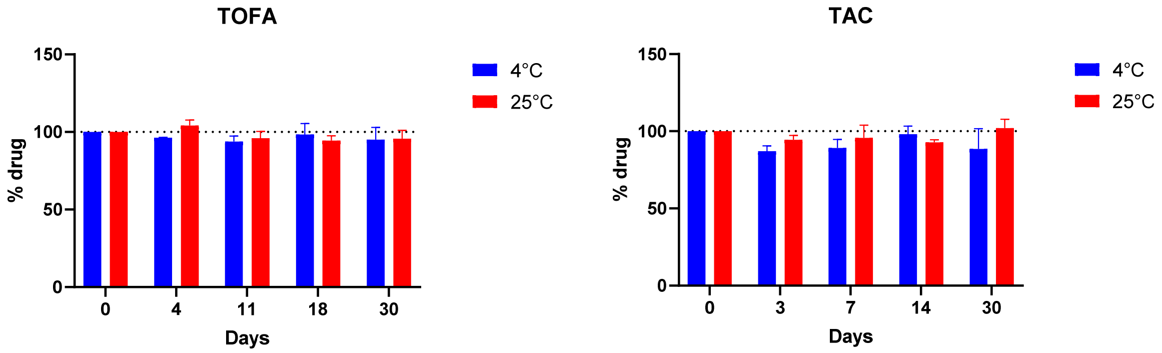

Supplementary figure 9. Long-term stability of TOFA loaded into TIF-gel and TAC loaded into TIF-gel over one month. Data are expressed as mean ± SD (n=3). Source data are provided as a Source Data file.

Stability study of TIF-Gel

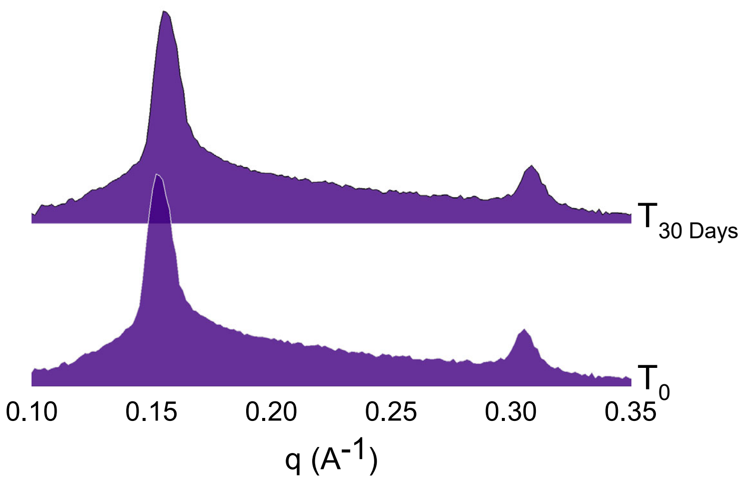

Supplementary figure 10. The lamellar geometry obtained immediately after the preparation of the gel does not change after 30 days of storage. *In vitro* characterizations of the TIF-Gel: SAXS spectra acquired at 25 °C immediately after the preparation (T_0) and after 30 days ($T_{30\ days}$). The calculated lattice parameters at T_0 and $T_{30\ Days}$ are 4.8 and 4.6 nm, respectively. The slight decrease in the measured lattice parameter can be explained by the loss of a minimal amount of water from the sample which was stored in a 2 mL microcentrifuge conical tube. Source data are provided as a Source Data file.

Dead volume of the syringe and cannula

To calculate the dead volume of the syringe 1 ml: Injekt®-F (Fine Dosage) Luer Solo (Luer Slip) (Braun) with the canula (size 20G, L × diam. 1.5 in. × 1.9 mm), the syringe was filled with different amount of formulation MLO + MilliQ water (84% lipid and 16% water) and the amount that came out of the syringe was recorded.

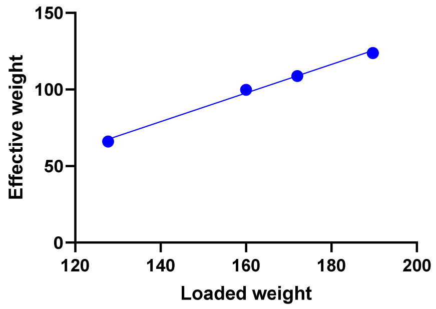

Supplementary figure 11. Calibration curve used to calculate the amount of formulation that has to be loaded into the syringe to obtain exactly 100 mg of formulation from the syringe with the cannula. Source data are provided as a Source Data file.

LC-MS/MS analysis

Samples, standards and QC were extracted by protein precipitation and analyzed by LC-MS/MS using the following method. For plasma samples, 10µL of plasma was mixed with 25 µL of precipitation solution (80:20 Acetonitrile: Methanol + 0.1 µM loperamide). The samples were centrifuged at 10000g for 10 minutes and 20µL of supernatant was diluted with 40µL of H_2O+0.1%FA. The samples were centrifuged at 3400rpm for 10 minutes and 50µL of supernatant was diluted with 100µL of H2O+0.1%FA. All the samples were analyzed by LC-MS/MS (Shimadzu prominence HPLC coupled to an AB/SCIEX 4000 QTRAP) in positive MRM mode. The samples were separated on a Cortecs RP shield column (3x50mm 2.6u) using a fast gradient of 10 mM ammonium formate in water (A) and methanol (B). The gradient starts at 20%B and increases to 98%B in 2 minutes, hold for 0.5 minutes and equilibrates for 1.4 minutes. The MRM parameters were optimized for each analyte; the MRM transition 313. to 149.3 was selected for TOFA, 822.3 to 770.1 for TAC and 477.1 to 266.0 for loperamide (internal standard). The samples were quantified using a calibration curve prepared in matrix using the area ration of analyte to internal standard.
