# Supplementary material for: Temperature-triggered in situ forming lipid mesophase gel for local treatment of ulcerative colitis
Source: Nat Commun. 2023 Jun 13;14:3489. doi: 10.1038/s41467-023-39013-3 (PMC10264425; doi:10.1038/s41467-023-39013-3)
Supplement: Supplementary file 3 — Reporting Summary [file 41467_2023_39013_MOESM3_ESM.pdf]

Corresponding author(s): Philippe Krebs
Gerhard Rogler
Paola Luciani
Simone Aleandri

Last updated by author(s): Apr 6, 2023

Reporting Summary

Nature Portfolio wishes to improve the reproducibility of the work that we publish. This form provides structure for consistency and transparency in reporting. For further information on Nature Portfolio policies, see our Editorial Policies and the Editorial Policy Checklist.

Statistics

For all statistical analyses, confirm that the following items are present in the figure legend, table legend, main text, or Methods section.

n/a	Confirmed	
☐	☒	The exact sample size (*n*) for each experimental group/condition, given as a discrete number and unit of measurement
☐	☒	A statement on whether measurements were taken from distinct samples or whether the same sample was measured repeatedly
☐	☒	The statistical test(s) used AND whether they are one- or two-sided<br>*Only common tests should be described solely by name; describe more complex techniques in the Methods section.*
☐	☒	A description of all covariates tested
☐	☒	A description of any assumptions or corrections, such as tests of normality and adjustment for multiple comparisons
☐	☒	A full description of the statistical parameters including central tendency (e.g. means) or other basic estimates (e.g. regression coefficient) AND variation (e.g. standard deviation) or associated estimates of uncertainty (e.g. confidence intervals)
☐	☒	For null hypothesis testing, the test statistic (e.g. *F*, *t*, *r*) with confidence intervals, effect sizes, degrees of freedom and *P* value noted<br>*Give P values as exact values whenever suitable.*
☐	☒	For Bayesian analysis, information on the choice of priors and Markov chain Monte Carlo settings
☐	☒	For hierarchical and complex designs, identification of the appropriate level for tests and full reporting of outcomes
☒	☐	Estimates of effect sizes (e.g. Cohen's *d*, Pearson's *r*), indicating how they were calculated

Our web collection on statistics for biologists contains articles on many of the points above.

Software and code

Policy information about availability of computer code

Data collection	GraphPad Prism 9.3.1.471., FACSDIva v6, ZEN imaging software3.7, Living Image® software v4.7
Data analysis	GraphPad Prism 9.3.1.471. , FkowJo V10.8, caseviewer 2.4, Microsoft Excel

For manuscripts utilizing custom algorithms or software that are central to the research but not yet described in published literature, software must be made available to editors and reviewers. We strongly encourage code deposition in a community repository (e.g. GitHub). See the Nature Portfolio guidelines for submitting code & software for further information.

Data

Policy information about availability of data

All manuscripts must include a data availability statement. This statement should provide the following information, where applicable:

- Accession codes, unique identifiers, or web links for publicly available datasets
- A description of any restrictions on data availability
- For clinical datasets or third party data, please ensure that the statement adheres to our policy

The datasets generated during and/or analysed during the current study are available from the corresponding author on reasonable request. A data table with values from all experiments has also been provided

Human research participants

Policy information about studies involving human research participants and Sex and Gender in Research.

Reporting on sex and gender	N/A
Population characteristics	N/A
Recruitment	N/A
Ethics oversight	N/A

Note that full information on the approval of the study protocol must also be provided in the manuscript.

Field-specific reporting

Please select the one below that is the best fit for your research. If you are not sure, read the appropriate sections before making your selection.

☒ Life sciences ☐ Behavioural & social sciences ☐ Ecological, evolutionary & environmental sciences

For a reference copy of the document with all sections, see nature.com/documents/nr-reporting-summary-flat.pdf

Life sciences study design

All studies must disclose on these points even when the disclosure is negative.

Sample size	Using a statistics software in experimental study design (G*Power), we calculated that in order to have at least 25% difference between the means, with a standard deviation of 10% (estimated from our previous proof of concept study) , we should have 90% power size with n=6 mice/group
Data exclusions	No data were excluded from analysis
Replication	All attempts of replications were successful. No replicates of animal data are used in the study.
Randomization	Mice were ear-marked, randomly assigned to different cages and treatment groups, and bedding mixed between all cages to avoid potential cage effects on microbiota.
Blinding	Histopathology scoring was performed by board-certified pathologist, in a blinded manner. Mice were randomized in each cage for the TOFA experiments, but true blinding was not possible as the compounds in the syringes are apparent.

Reporting for specific materials, systems and methods

We require information from authors about some types of materials, experimental systems and methods used in many studies. Here, indicate whether each material, system or method listed is relevant to your study. If you are not sure if a list item applies to your research, read the appropriate section before selecting a response.

Materials & experimental systems

n/a	Involved in the study	
☐	☒	Antibodies
☒	☐	Eukaryotic cell lines
☒	☐	Palaeontology and archaeology
☐	☒	Animals and other organisms
☒	☐	Clinical data
☒	☐	Dual use research of concern

Methods

n/a	Involved in the study	
☒	☐	ChIP-seq
☐	☒	Flow cytometry
☒	☐	MRI-based neuroimaging

Antibodies

Antibodies used	T cells (defined as CD3+ cells; antibody used: eBioscience, 25-0031-82, dilution in PBS: 1:200); dendritic cells (CD11c+, CD11b+; Biolegend 117324 & 101241, dils 1:400, 1:200); neutrophils (CD11b+; Ly6G+; Biolegend, B156884, 1:100), macrophages (CD11b+, CD11c-, Ly6G-, Ly6Clo), inflammatory monocytes (CD11b+, Ly6Chi; Biolegend, 128024, 1:100), and eosinophils (CD11b+, CD11c-,

	Ly6G-, Siglec F+; BD Bioscience 552126; 1:100)

Validation

Validations statements are on the manufacture' s web site BD biosciences states: The specificity is confirmed by using multiple applications that may include a combination of flow cytometry, immunofluorescence, immunohistochemistry or western blot to test a combination of primary cells, cell lines or transfectant models. All flow cytometry reagents are titrated on the relevant positive or negative cells. To save time and cell samples for researchers, pre-titrated test size reagents are bottled at an optimal concentration, with the best signal-to-noise ratio on relevant models. You can look up the Certificate of Analysis and the concentration of test-size human reagents from specific lots via the Concentration Lookup page or BD Regulatory Documents
Biolegend states:
Antibody validation is a critical step in the journey towards obtaining consistent reproducibility in science. To ensure they are both specific and sensitive, we validate our antibodies through a variety of methods including:

Testing on multiple cell and tissue types with a variety of known expression levels.
Validation in multiple applications as a cross-check for specificity and to provide additional clarity for researchers.
Comparison to existing antibody clones.
Using cell treatments to modulate target expression, such as phosphatase treatment to ensure phospho-antibody specificity.

ebioScience (invitrogen) states:
Invitrogen antibodies are currently undergoing a rigorous two-part testing approach
Part 1—Target specificity verification
This helps ensure the antibody will bind to the correct target. Our antibodies are being tested using at least one of the following methods to ensure proper functionality in researcher's experiments. Click on each testing method below for detailed testing strategies, workflow examples, and data figure legends.

Knockout—expression testing using CRISPR-Cas9 cell models
Knockdown—expression testing using RNAi to knockdown gene of interest
Independent antibody verification (IAV)—measurement of target expression is performed using two differentially raised antibodies recognizing the same protein target
Cell treatment—detecting downstream events following cell treatment
Relative expression—using naturally occurring variable expression to confirm specificity
Neutralization—functional blocking of protein activity by antibody binding
Peptide array—using arrays to test reactivity against known protein modifications
SNAP-ChIP™—using SNAP-ChIP to test reactivity against known protein modifications
Immunoprecipitation-Mass Spectrometry (IP-MS)—testing using immunoprecipitation followed by mass spectrometry to identify antibody targets
Part 2—Functional application validation
These tests help ensure the antibody works in a particular application(s) of interest, which may include
(but are not limited to):

Western blotting
Flow cytometry
ChIP
Immunofluorescence imaging
Immunohistochemistry

Animals and other research organisms

Policy information about studies involving animals; ARRIVE guidelines recommended for reporting animal research, and Sex and Gender in Research

Laboratory animals	Female 6-8 week-old C57BL/6J mice were ordered from Charles River (Germany).<br>Rag-/- mice in the C57BL/6 background (originally purchased from Taconic from which a local colony was maintained in our vivarium)
Wild animals	This study did not involve wild animals
Reporting on sex	12-15 week-old male and female Rag-/- mice in the C57BL/6 background. 3 male and females/ groups.<br>Female 6-8 week-old C57BL/6J mice. 6 females/ group
Field-collected samples	This study did not involve sample collected from the field
Ethics oversight	All methods used were approved by the Cantonal Veterinary Offices of Berne and Zurich. Permission no. BE 20/18 and Permission no. ZH043/2021, respectively.

Note that full information on the approval of the study protocol must also be provided in the manuscript.

Flow Cytometry

Plots

Confirm that:

☒ The axis labels state the marker and fluorochrome used (e.g. CD4-FITC).

☒ The axis scales are clearly visible. Include numbers along axes only for bottom left plot of group (a 'group' is an analysis of identical markers).

☒ All plots are contour plots with outliers or pseudocolor plots.

☒ A numerical value for number of cells or percentage (with statistics) is provided.

Methodology

Sample preparation	Briefly, mouse spleens (after weighing) and mesenteric lymph nodes were homogenized through a 70 µm cell strainer, after which point the red blood cells were removed from the spleen by re-suspending the cell pellet in ACK lysing buffer (150mM NH4Cl, 10mM KHCO3, 0.1mM; pH: 7.4) at room temperature for 5 min. Splenocytes were quantified using a CASY cell counter (Omni Life Sciences) and the following populations were quantified following single cell and live/dead selection (Thermofischer, L34961).<br>Lamina propria cells were isolated as describe previously (Spalinger et al Mucosal Immunology, 2019, DOI: 10.1038/s41385-019-0201-1, cited in the manuscript)
Instrument	BD BioscienceLSR II SORP flow cytometer or a BD Biosciences LSR Fortessa
Software	Data were collected with the BD FACSDiva software and analyzed with FlowJo version 10.
Cell population abundance	Cells were not sorted or otherwise purified, only quantified. All of the relative abundances for the cells are noted in the manuscript: For spleen, absolute numbers are also provided. Sample purity was established at the organ level (i.e. resections of spleen and mLNs)
Gating strategy	The flow cytometry gating strategy was adapted from Liyanage et. al (2016) Exp. Eye res. PMCID: PMC5053376, as cited in the manuscript. Briefly, all candidate cells were gated by Forward and Side scatter, then T cells were selected by CD3e positivity. After this, cells were gated for CD11b and CD11c (double-positive population: DCs). CD11b+ cells were further used to gate for Ly6G. Positive cells were Identified as neutrophils. Negative cells were stained for Ly6C (high: infl. Monocytes, low-mid: macrophages. SiglecF was used for Eosinophils. For nearly all populations, except DCs, the boundaries of contour plots or histograms were used for delineating cell populations. DCs were not highly abundant and present as a scattered population, as is also seen in our cited gating strategy manuscript.<br>For Flow cytometry of T cell ransfer colitis, the gating was performed as described previously (Spalinger et al Mucosal Immunology, 2019, DOI: 10.1038/s41385-019-0201-1). Cells were gated based on forwar/side scatter, douplets excluded based on FSC-H/FSC-A, then gated on live CD45+ cells. In this subset, CD3+ cells were considered as T cells. On this gate, Tregs were defined as FoxP3+CD4+ cells, and Th1 (IFNg+) and Th17 (IL17+) cells were gated from CD4+ cells.

☒ Tick this box to confirm that a figure exemplifying the gating strategy is provided in the Supplementary Information.
